# Hospitals

**Published:** 1876-08

**Authors:** 


### Hospitals.

### MEDICAL DEPARTMENT, COUNTY HOSPITAL.

Service of Dr. Lyman.

(Reported by Dr. Fenn.)

May 2, 1876. Dr. Lyman presented and made remarks on the following cases:

1. *Chronic Bronchitis.*

Duration three years. A young man. Dyspnœa and cough, worse at night, owing to irritation of sympathetic sub-mucous ganglia. In the day time the influence of the brain reaches the ganglia and restrains them. At night the brain is asleep and the ganglia are left free to respond to excitement. This man's cough and dyspnœa have continued for two years. There is shortness of breath from diminished calibre of the bronchial tubes. In infants swelling narrows the small tubes more than in adults. In infants, therefore, the dangers from dyspnœa are much greater.

*Treatment.* Care in the acute stage, for there is danger of its becoming chronic. Allay nervous irritability; liquid food; divert the column of the blood; act on the capillary net work to dilate it and allow the blood to go on. Tartar emetic and sulphate of morphia of each $\frac{1}{32}$ of a grain every hour go well together. In a short time there will be copious perspiration. To promote expectoration give squills. Finally, if there be danger of the attack becoming chronic, administer benzoin, pitch and tar fumes; and later, and by all means if the attack have become chronic, iodide of potassium. This patient had it; whenever it was omitted he got worse. He will probably be an invalid as long as he lives, or at least liable to a new attack with every exposure to cold.

May 5. A number of cases illustrative of diseases of the liver.

1. *Acute Cirrhosis.*

A bright, good looking young woman, aged twenty-two, from the dispensary. Dr. L. remarked that here was a case illustrating the effect of acute disease of the liver. There is a considerable degree of prominence to the abdomen. The lower portion is filled with fluid. Chest resonance lost on the right over the nipple. Dullness on the left. Back dull at a point just below the scapula. The ear applied to the chest detects a respiratory murmur only above the point of dullness. Her hands are cold; tongue red and fissured; feet slightly swollen. Her life has not always been what it should be; she has had syphilis. On her recovery from this she resumed her old course, but had greater hardships. A year ago she began to complain of pain in the right side. She began to take morphia and has continued it ever since. This perhaps accounts for the condition of the tongue ; these fissures always indicate chronic dyspepsia. Coldness of the hands is accounted for by the habit of taking opium. Now how shall we account for the enlargement of the abdomen? She says that three weeks ago she was feeling pretty well; then she began to have severe pain under the short ribs, where her pain began a year ago. The dullness extends to the limit of an enlarged liver. In this young person we have a cause which is apt to produce inflammation in any or every organ of the body. Syphilis when it attacks the liver produces degeneraton; obliteration of vessels ensues. This is as if an obstruction occurred in the current of a river; it may be the product of inflammation of Glisson's capsule. The watery portion of the blood accumulates by extravasation into the abdominal cavity. Two years ago syphilis; one year ago pain; now syphilitic disease of the liver. We only wish this might be the last link in the chain of evils that follow. She will probably return for tapping. Dr. L. recalled the anatomy of the liver and recommended to get the idea in a bunch of grapes. The stalk is the hepatic vein. A spider spins his web amid the clusters. This may represent the ramifications of the hepatic artery. Another spider's web over the same surface may represent the portal veins in their finest distribution. The lymphatics and nerves in like manner intimately pervade the spaces between the lobules. Another system or web exists in the bile ducts themselves ready to receive the bile, one of the most complicated and most beautiful. All these elements are supported by areolar tissue. Here is the seat of this inflammation. First there is swelling, then effusion, then contraction; hence pressure interfering with the passage of blood, with nutrition of the liver, and with the removal of bile — in every respect like a cluster of grapes, shrinking until it becomes a dry and sapless bunch of raisins. This condition exists in the following case:

2. *Chronic Cirrhosis.*

Admitted March 20th. The abdomen is sunken; great emaciation. On percussion of the lungs resonance is great; resonance where it should be dull; that is, over the region of the liver. Resonance all over the abdomen in varying degree, according to the size of the bowel. The liver is evidently shrunken. A drop of sulphuric acid in a little of his urine, on a plate, develops a violet color which spreads, showing bile to be in the urine. The only way of escape to the bile, if any hepatic cells retain the power of secretion, is by resorption into the blood vessels. This is cirrhosis. There is no fluid in this case because the man has hemorrhoids. The bloody discharges unload the engorgement in a measure; besides, we believe that anastomosis has taken place between these enlarged hemorrhoidal vessels and the internal iliac. He also has diarrhœa. There is distention of the capillaries and thickening of the mucous lining of the stomach and intestinal canal. Nutrition is interfered with. The patient starves to death. The temperature is elevated to 99½°, enabling us to distinguish these from cancerous affections, in which the temperature is usually depressed. He is really in the last stages.

3. *Chronic Cirrhosis; Dropsy.*

Another old case exhibited as a variety of the disease. He has been tapped twice. Now the distension that has again occurred demands that the operation be repeated. Note particularly the effect upon the umbilicus, where it appears as if there was a little hernia. This is owing to the weaker structure of the abdominal walls at this point from the great pressure of the fluid within. The most common cause of cirrhosis of the liver is alcohol. Alcohol promotes a tendency to the coagulation of albumen. As soon as it is all absorbed it has to pass through the liver. The brunt of the attack is here; constant irritation does the work. The disease is difficult to trace clear to its origin, but little by little it comes on. Finally there is almost complete disappearance of the channels through the liver, as we have described; outpouring of serum and plastic lymph into the cavities or in the areolar tissues of the body. The duration is essentially chronic, but if the cause be syphilis it is apt to be rapid; little can be done. It cannot be cured; we can only palliate; draw off fluid; relieve pain; assist bowels, urinary organs and skin. In general, sooner or later, the patient becomes a victim to the anatomist's scalpel.

1. *Typhoid Fever.*

May 9. There is the same relation between spurious and genuine typhoid fever that exists between cholera morbus and Asiatic cholera. The lesion is the same when death occurs, but there will be no propagation of the disease. True typhoid fever is propagated. A great amount of the typhoid fever in towns is due to contamination of the soil and percolation into the wells and springs. It is a very important thing, therefore, to be careful what becomes of the excretions of typhoid patients. Dr. L. related an account of the outbreak of typhoid fever which recently occurred at the village of Lausanne, in Switzerland. (*Nature*, Vol. xiii., p. 447.) The source of the poison was traced to an isolated farm house on the opposite side of a mountain ridge, where an imported case of typhoid fever followed by two others occurred shortly before the outbreak. A brook which ran past this house received the dejections of the patients, and their linen was washed in it. This brook was employed for the irrigation of some meadows near the farm house, and the effluent water filtered through the intervening mountain to a spring used in all the houses of Lausanne except six which were supplied with water from private wells. In these six houses no case of fever occurred, but scarcely one of the others escaped. No less than one hundred and thirty people, or seventeen per cent. of the whole population were attacked besides fourteen children who received the infection whilst at home for their holidays and afterwards sickened on their return to school. The passage of water from the irrigated meadows to the spring at Lausanne was proved by dissolving in it at the meadows eighteen hundred weight of common salt and then observing the rapid increase of chlorine in the spring water, but the most important and interesting experiment consisted in mixing uniformly with the water fifty hundred weight of flour, not a trace of which made its way to the spring, thus showing that the water was *filtered* through the intervening earth and did not pass by an underground channel. So far as medicine is concerned there is very little to do. We have the effect of poisoning of the blood, interference with nutrition and with elimination. It requires a determinate time for these changes to take place. Sometimes eliminants are sufficient; but generally the germs have propagated themselves and have poisoned the tissues. The tendency is to destruction of tissue. The temperature here now is 103°.

We must supply nutriment; food should be given frequently and in small quantity. The diarrhœa in the early stages of the disease is due to irritation of the mucous lining of the stomach. It is not well to interfere in the first stage; but in later stages give acids. Mineral acids have no direct curative effect; secure sleep. Be careful about the administration of opiates. Do not leave them to the discretion of the nurse, lest they produce a tendency to collapse. The temperature should be attended to above everything else. The heart becomes degenerated very rapidly by the great heat of the blood; hence the feebleness. The patient may faint and sudden death may occur. The effect of the high temperature on the nervous system is to produce delirium. To lower the temperature of the patient put him in a bath at the temperature of the blood, then cool to 72°. Keep him in ten minutes. Afterwards watch him; if his temperature rise to 102½° put him in the bath again. This treatment has reduced mortality greatly; it diminishes the dangers from every direction. In the epidemic which occurred among the dregs of European armies after the Franco-Prussian war, the mortality was reduced from twenty-seven per cent. to eight per cent. by this method. Some precautions are very necessary; use the thermometer; have a good nurse, and follow up faithfully. After putting the patient back to bed, a little wine may be given if he is faint. Thus typhoid fever becomes an easy and agreeable disease to treat.

2. *Pneumonia, complicated with Pleuritis, Bronchitis and Tubercular Deposits.*

A fair-skinned young man pretty well developed, arrived yesterday. He has had a cough for three years, as he says. One week ago, after sitting in a cold draft, he had a chill and great pain in the right side. Respiration now 56; temperature 104°; tongue normal; expectoration frothy, tenacious, colored with blood. Such sputa are complex. The frothy portion is from the bronchial tubes. The tenacious portion arises in the bronchioles; the blood is partly from there, but primarily from the air cells of the lungs. Resonance exists all over the left; dullness all over the right; loss of respiratory sound at the base. Over the middle it is almost cavernous, so exaggerated is the tubular character of the breathing. There is also a well marked friction sound anteriorly. Rhonchus is perceptible to the hand on both sides. We have a combination here of inflammation of the air cells and finer air tubes, pleurisy and bronchitis; this patient has had a cough for three years. Since it is unusual to find pneumonia at the apex without tubercular deposit, it is probable that we have tuberculosis also. But in tuberculosis we have have no characteristic symptom. In pneumonia the exudation is in the air cells and in the bronchioles. The consolidation is the same; the irritation is the same. But a distinction may be certainly derived by watching the location and progress of the consolidation. It is not probable that he will get well perfectly, but rather that the tuberculosis will extend. We can only assure him of an amelioration of his symptoms. The man will get back with another attack in a year or two. Treatment rational. He has only one lung to breath with now; hence there is an accumulation of carbonic acid in the blood. Make the blood flow more rapidly by inviting it from the lungs to the surface and relieve the pressure in the engorged capillaries. Mustard and cups will do this. The lung surface equals 200 square yards of aerating membrane. In this case 100 square yards are gone. Supply the deficiency by increasing the activity of the bowels, skin and kidneys. A stimulating cathartic, as of rhubarb, soda and calomel, will do well. Give acetate of potash water, or effervescing powder and water to stimulate the kidneys; stimulating expectorants; give ipecac., just short of producing nausea, often as once in two or three hours, small doses often. If patient get relief from poultices use them. If he be a child, use oil silk lined with Canton flannel; it is almost as good as a poultice and neater. Pour in nourishment as rapidly as possible; milk, milk-punch, stimulants in large quantities; sometimes a quart of brandy daily; even two quarts in twenty-four hours; but in general a tablespoonful every hour is sufficient. Avoid opiates, for they interfere with the circulation of the blood by contracting the capillaries. Resort to remedies which dilate the capillaries; chloral does this.

---

### REPORT OF CLINICS IN COUNTY HOSPITAL.

Medical Department, Service of Dr. Bevan.

(Reported by Dr. Fenn.)

*The Cook County Hospital for the Month ending May* 10, 1876.

March 21. Dr. Bevan introduced and made remarks on the following cases:

1. *Locomotor ataxia.*

A fair, black haired woman, aged about forty. There is loss of ability to use the limbs; simply want of power of co-ordination. She is able to resist flexion or extension, when the attempt is made passively, to bend or to straighten her limbs against her will. The capacity of man's strength is inherent in the muscles themselves; but if this patient attempts to stand with her eyes shut she totters over. There is also a great amount of confusion in her head if she merely shuts her eyes lying down; vertigo, dizziness. Pathological anatomy discovers changes in the spinal cord, a low degree of hyperæmia, known in *post mortems* by degeneration of the posterior columns; sometimes sclerosis, sometimes softening or thickening. These patients seldom die but of some intercurrent disease, enteritis, disease of the bladder or some lung affection.

*Treatment.* Nothing permanently influences it. Counter irritation is of no use. All forms have been tried, even the actual cautery. Sometimes good seems to be derived from the neuro tonics. Sometimes from electricity, by keeping the nerves to the lower extremities somewhat stimulated. On the theory that there is hyperæmia, the treatment mentioned in the history was bad. It will probably all fail.

2. *Tubercular degeneration of the mesenteric glands.*

A pale, emaciated child, aged five or six years; has been sick four months. He has had a cough continually. Spits white thick stuff. Abdomen rather portuberent, as in scrofula. Dullness at the apex of the left lung.

*Treatment.* Two drachms of cod liver oil and fifteen drops of the syrup of iodide of iron. Externally paint with the tincture of iodine and apply oil silk or chamois skin.

1. *Pathological.*

March 25. Exhibited a brain from a man who had died of apoplexy. Duration one week. A physician. On laying open the ventricles, a large dark clot of blood was found in the left and a collection of serum in the right. The condition of the tissue over the clot was that of disorganized brain matter, diffused like a layer of mortar. The surface of the brain was covered with a thin, pearly coating, the result of fibrinous effusion. Under the membrane a decided serous effusion was found, which flowed away. The paralysis had been so great that he could neither swallow food nor medicine.

Dr. B. remarked that the Hospital afforded numerous such cases, but the friends generally desire to take them away, hence the opportunities for *post mortem* examination are few.

2. *Chronic Eczema capitis.*

Old man, aged fifty-three. Disease since last August. Moisture has about ceased.

*Treatment.* Add six grains of carbolic acid to each ounce of the benzoated oxide of zinc ointment as a stimulant, and give ten drops of Fowler's solution three times daily.

3. *Sciatica.*

Of moderate intensity. The man is from Riverside, where they have ague. A blister has been directed for the outside of the trochanter, and the nerve will be followed up with counter irritants. Also six grains of quinine to be given four times daily, and two grains of opium at night.

1. *Epilepsy.*

March 28. Twelve years standing. Young woman, aged 26, black haired. Opisthotonos is so great during the attack that her body describes half a circle. There is an amount of rigidity that leads almost to muscular inflammation. Soreness continues for some time. The face is seen to be twitching: she says she feels the spasms working on her now. She complains that her flesh feels as if it was full of little needles. She has been receiving thirty grains of bromide of potassium four times daily, and recently one grain of belladonna. It is now proposed that she have an increase of half a grain every five days until she gets three grains at a dose. It affects her sight. The bromide will be continued.

2. *Scurvy.*

A well developed young Norwegian.

*Treatment.* Abundance of nourishment.

3. *Chronic Dysentery.*

Eight months. An emaciated, slight man, aged about thirty. Seven stools daily. Loss of appetite. Ordered for him yesterday one grain each of opium and ipecac., and two grains of camphor, three times daily. There is entero colitis. Strong nitric acid has been applied to the surface lately, as high as possible, with benefit. Milk diet. The disease is hard to cure here. The class of patient is unfavorable. The food is unfavorable.

